# Two Cases of Traumatic Tetanus Successfully Treated by Ice

**Published:** 1853-12

**Authors:** 


In introducing this article to the notice of our readers, we call attention to the fact that it occurred upon Long Island, a locality where traumatic tetanus is exceedingly common among both men and horses. Long Island alone could probably furnish a larger list of cases of tetanus than any other district, of ten times its population, in this country. S. B. H.

*Two cases of Traumatic Tetanus successfully treated by Ice.* By B. D. Carpenter, M. D., Cutchogue, Suffolk Co., Long Island.

Case I. August 22d, 1819.—E. G., aged 16 years, of good constitution and habits, jumped from a fence on the stump of a twig some half inch in diameter; which made a wound in the ball of the right foot ¾ of an inch deep. Twelve days after the accident he complained of feeling lame and stiff, during the night was awaked by a violent spasm; the next day complained of stiffness and soreness of the muscles of the neck and throat, and pain at the scrobiculus cordis; the following night, during sleep, was seized again with spasm; and the next morning when I was sent for, I found him complaining of pain in the above region, great rigidity of the whole muscular system, attended with difficulty in swallowing and constraint in moving the head and jaws, and in articulating. During the spasm, the body was curved backward and thrown to one side, the dyspnœa was considerable, pulse full and slightly accelerated, skin warm and moist, bowels costive, urine scanty and high-colored.

Administered a purgative, which was assisted by enemas. The patient was then put upon the free use of opium in the shape of Dover's powder, and the bowels kept open by the use of cathartics and injections of ℥i. tinct. assafœtida in half a pint of soap suds, repeated as often as the preceding one came away. This treatment was continued for four days, during which time he gradually grew worse. The tetanic rigidity and spasm increased until the sixth day; when, finding that he must die unless something farther could be done to allay the pain and extreme spasm, and viewing the difficulty as being an irritation of the spine, perhaps connected with congestion of the membranes covering the spinal marrow, I determined to apply ice to the head and the whole length of the spinal column, since the whole muscular system was affected. I did so, and in ten minutes had the satisfaction of seeing the pulse come down from 110 to 75, and all the urgent symptoms relieved; the rigidity was gone, and he had but one spasm after the ice was applied; his bowels were kept open, and assafœtida injections were continued twice a day, to allay the irritability of the nervous system, manifested by slight twitchings. No medicines were given by the mouth. The wound entirely healed, and in three days the patient was discharged cured; and his health since has been as perfect as before the attack.

Case II. August 11th, 1853.—A. C., 21 years of age, a robust farmer, in good health, in assisting to remove some old lumber, stepped on the point of a rusty nail, which entered the hollow of the foot to the depth of ¾ of an inch. The wound was not very sore, and was dressed with some simples by himself; and he remained at work moderately until the 16th, five days after the accident, when he complained in the afternoon of twitching in that foot and slight pain in the region of the wound and leg of that side. Was quiet the rest of the day, and retired early to bed, but slept none from restlessness, anxiety, and slight pains and twitching of the nervous system. On the 17th felt some pain in the head and through from the lower end of the sternum to the back. I saw him at 6, P. M., and found him complaining of pain as above mentioned, which had gradually increased at the sternum, great rigidity of the muscles of the left side of the neck, accompanied with slight dyspnœa and some difficulty in swallowing. Even at this time there was present the peculiar expression of countenance found in tetanus. Pulse 100 and hard, bowels costive—had eaten nothing—the wound had not commenced to heal, and was covered slightly with a thin serous discharge. Made a free incision into the wound, and dressed it with bread and milk poultice, to which tinct. opii was added; ordered 10 grs. of calomel with 10 of rhei, to be followed by pil. colocynth. comp until the bowels were freely moved, and enemas of tincture of assafœtida, ℥j. every three hours, or as often as the preceding one should be voided, large doses of Dover's powder by the mouth, and to have the neck bathed in camphorated oil and tinct. opii. 18th, 7, A. M., found that the bowels had been freely moved and that spasm of the whole muscular system had commenced. About 3 o'clock, A. M., pain in the neck and at the sternum increased, and there was great rigidity of the muscular system generally; dyspnœa great, much difficulty in swallowing and articulation, jaws partially closed, entirely so during the spasm, pulse 120; indeed all the symptoms increased in a marked degree, with slight delirium. Ordered ¼ of a grain of morphine every hour, and to continue the assafœtida injections. 6, P. M., all the symptoms greatly aggravated, pulse so small and frequent that it could not be counted, jaws closed, breathing extremely difficult, body almost constantly drawn backward or forward and to one side, face pale, skin moistened with clammy sweat, and perfect rigidity of muscular system. Had slept none for forty-eight hours. Applied ice to the head and whole length of spinal column; in twenty minutes the pulse came down to 100, the skin was covered with profuse perspiration, the muscular system relaxed; in short there was a perfect yielding of all the urgent symptoms, and the patient slept soundly and pleasantly for the succeeding two hours, during which time the breathing was natural, and there was neither tetanic rigidity or spasm. When he awoke, there was still some delirium, the pain in the region of the sternum was very great, and for half an hour the tetanic rigidity and spasm were considerable. The ice was again applied, when the symptoms immediately yielded, and the patient (with the exception of short intervals) slept quietly the balance of the night.

17th. 6, A. M., the bowels were moved by the assafœtida injections, the delirium had passed off, all the tetanic rigidity was gone. Pulse 80, breathing natural, but said there was great soreness of the chest and all the muscles of the body. Drank some soup, continued the ice and injections as before. 11, A. M., there was some slight twitching of the muscles, without rigidity; from this time the patient continued to improve without either tetanic rigidity or spasm until, on the 25th, he was discharged cured, with the wound nearly healed.

The ice was applied from ten to thirty minutes each time, with intervals of from two to eight hours.—*New York Medical Times.*

